# Physical Properties of CaTiO_3_-Modified NaNbO_3_ Thin Films

**DOI:** 10.3390/nano14141186

**Published:** 2024-07-12

**Authors:** Yongmei Xue, Li Ma, Zhuokun Han, Jianwei Liu, Zejun Wang, Pengcheng Liu, Yu Zhang, Huijuan Dong

**Affiliations:** 1 Department of Physics, Changzhi University, Changzhi 046011, China; 12022833@czc.edu.cn (Y.X.); mali9001@126.com (L.M.); ljw75_2002@163.com (J.L.); zejunwangcz@foxmail.com (Z.W.); 2 School of Science and Technology, Northwestern Polytechnical University, Xi’an 710072, China; hanzk@nwpu.edu.mail.com; 3 Research Institute of Opto-Mechatronics Industry, Jincheng 048000, China; 4 Shanxi Key Laboratory of Advanced Semiconductor Optoelectronic Devices and Integrated Systems, Jincheng 048000, China; zhangyu@semi.ac.cn

**Keywords:** lead-free, NaNbO_3_, physical properties

## Abstract

NaNbO_3_(NN)-based lead-free materials are attracting widespread attention due to their environment-friendly and complex phase transitions, which can satisfy the miniaturization and integration for future electronic components. However, NN materials usually have large remanent polarization and obvious hysteresis, which are not conducive to energy storage. In this work, we investigated the effect of introducing CaTiO_3_((1−*x*)NaNbO_3_-*x*CaTiO_3_) on the physical properties of NN. The results indicated that as *x* increased, the surface topography, oxygen vacancy and dielectric loss of the thin films were significantly improved when optimal value was achieved at *x* = 0.1. Moreover, the 0.9NN-0.1CT thin film shows reversible polarization domain structures and well-established piezoresponse hysteresis loops. These results indicate that our thin films have potential application in future advanced pulsed power electronics.

## 1. Introduction

Perovskite oxides exhibit a diverse range of physical properties, such as ferromagnetism, antiferroelectricity and superconductivity, which have garnered significant attention from researchers, making the comprehension of the composition–structure–performance relationship in antiferroelectric (AFE) materials a prominent research focus within the field of dielectric materials. Antiferroelectric perovskite oxides can realize reversible or irreversible transitions between antiferroelectric and ferroelectric states under electric field excitation, accompanied by substantial changes in charge, volume, or heat. Thus, they have considerable potential for applications in high-density energy storage devices and electromechanical transducers. Compared to many other lead-free AFE materials, lead-free sodium niobate (NaNbO_3_, NN) has great potential for energy storage applications due to its relatively wide band gap, low cost [1,2,3,4,5,6] and complex phase transitions over a wide temperature range [7,8,9,10,11,12]. However, NN tends to exhibit field-induced metastable ferroelectric, resulting in square rather than double *P-E* hysteresis loops. Nevertheless, the physical properties of NN-based materials are comparable to Pb-based dielectric materials [13,14,15]. For example, Luo et al. [13] achieved both a high recoverable energy storage density of about 19.64 J/cm^3^ and excellent thermal stability in 1 mol% Mn NNCZ thin film. To stabilize the AFE phase, researchers introduced a low tolerance factor (τ) or low polarizability comchalcogenide phase (ABO_3_) to form a NaNbO_3_-ABO_3_ solid solution system. Recently, Xie et al. [16] reported that (1−*x*)NaNbO_3_-*x*CaTiO_3_ solid solutions demonstrated outstanding comprehensive energy-storage properties, which have giant W_rec_ = 6.6 J/cm^3^, high energy efficiency η = 80% and ultrahigh power density P_D_ = 350 MW/cm^3^.

CaTiO_3_ (CT) is a linear dielectric material with characteristics of high dielectric constant, low dielectric loss and wide band gap [17,18,19]. It has been demonstrated by researchers that the introduction of CT into perovskite ceramics, e.g., SrTiO_3_ (ST) or AgNbO_3_ (AN), can effectively increase E_b_ and enhance energy storage performance. Wang et al. [20] pointed out that the Ca_0.5_Sr_0.5_Ti_0.9_Zr_0.1_O_3_ ceramic obtained a maximum breakdown strength of 390 kV/cm, a high energy storage density of 2.05 J/cm^3^ and an ultra-high energy efficiency of 85%. Xu et al. [21] showed that the incorporation of CaTiO_3_ could enhance the stability of AgNbO_3_ antiferroelectricity and a high recoverable energy density of 3.7 J/cm^3^ was obtained in 2 mol% CaTiO_3_-doped AgNbO_3_ ceramics. The above studies show that CT is an ideal candidate for linear energy storage thin films. However, the tolerance factor of NN (~0.967) is relatively low, and the difference in values with CT (~0.966) is negligible, favoring a better selection of the optimal composition [16]. Compared to bulk ceramics, thin films have higher electric breakdown strength and energy storage density, and thus have a broad application prospect in the field of energy storage capacitors.

We systematically investigated the physical properties of CaTiO_3_-modified NaNbO_3_ thin films in this work. The (1−*x*)NN-*x*CT (*x* = 0, 0.1, 0.2) of thin films were grown on (111) Pt/Ti/SiO_2_/Si substrates by the sol–gel method. Introducing smaller radii of Ca^2+^ and Ti^4+^ at A/B sites to improve the electrical properties of NN thin films, resulting in obtaining smaller grain sizes and a lower number of oxygen vacancy defects and making it more favorable for energy-related applications.

## 2. Experimental

**Figure 1:**
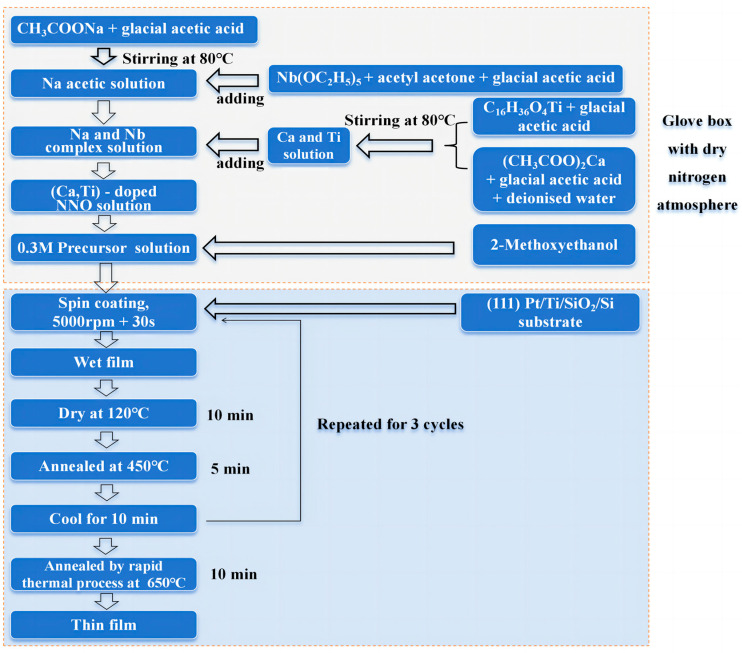
Preparation process for (1−*x*)NN-*x*CT thin films by sol-gel method.

The (1−*x*)NaNbO_3_-*x*CaTiO_3_ ((1−*x*)NN-*x*CT, *x* = 0, 0.1, 0.2) thin films with a thickness of approximately 200 nm were fabricated on (111) Pt/Ti/SiO_2_/Si substrates by the sol–gel method. Three precursor solutions with different CaTiO_3_ ratios were employed to obtain the NN thin films. The detailed preparation process is shown in Figure 1. High-purity sodium acetate [Na(CH_3_COO)_2_], niobium ethoxide [Nb(OC_2_H_5_)_5_], calcium acetate [Ca(CH_3_COO)_2_·H_2_O] and tetrabutyl titanate [Ti(OCH_2_CH_2_CH_2_CH_3_)_4_] were used as raw materials. The solvents included 2-Methoxyethanol (CH_3_OCH_2_-CH_2_OH), acetic acid (CH_3_COOH) and deionized water, while acetylacetone (CH_3_COCH_2_COCH_3_) served as a chelating agent. All the chemicals were purchased from Sigma-Aldrich (Merck KGaA, Darmstadt, Germany). The raw materials should be weighed according to the ratios, and a 10 mol% excess of sodium was added to the precursor solution to compensate for the sodium loss during the thermal process. Firstly, sodium acetate was dissolved in acetic acid, heating and stirring at 80 °C for 30 min. A mixture of niobium ethoxide and acetylacetone was dissolved in acetic acid, where the molar ratio of niobium and acetylacetone is 1:1. Secondly, calcium acetate was dissolved in acetic acid and deionized water and tetrabutyl titanate was dissolved in acetic acid, both at 80 °C with heating and stirring. Then, the above solutions were cooled to room temperature and mixed together to form a mixed solution of Na, Nb, Ca and Ti. Finally, 2-Methoxyethanol was added to the solution and the solution should be set to 2 mL. It should be noted that the entire preparation of the solution was carried out in a glove box filled with dry nitrogen at a precursor solution concentration of 0.3 mol/L. The solution was aged at least 48 h before preparing the thin films. Precursor solutions were spin-coated on (111) Pt/Ti/SiO_2_/Si substrates to form uniform wet films. The rotation speed was fixed at 5000 rpm and the spin time lasted 30 s. Each layer of NN wet film was initially dried at 120 °C for 10 min to evaporate the solvent, and then the process was repeated three times. Finally, the thin films were annealed at 650 °C for 5 min to obtain NN thin films with different CT contents.

X-ray diffraction (XRD, Panalytical Empyrean, Almelo, The Netherlands) and atomic force microscopy (AFM, Bruker-Demension Icon, Ettlingen, Germany) were used for structural and morphological characterization. The thickness of the film and elemental compositions were determined by scanning electron microscopy (SEM, FEI, Verios G4, Hillsboro, OR, USA) and analyzed by X-ray photoelectron spectroscopy (XPS, Thermo Scientific ESCALAB 250Xi, Waltham, MA, USA) with Al K radiation, respectively. Additionally, the dielectric properties to analyze by an Agilent E4980 LCR meter (Santa Clara, CA, USA), amplitude hysteresis loops and local phase were characterized by an MFP-3D atomic force microscope (Asylum Research, Santa Barbara, CA, USA).

## 3. Results and Discussion

**Figure 2:**
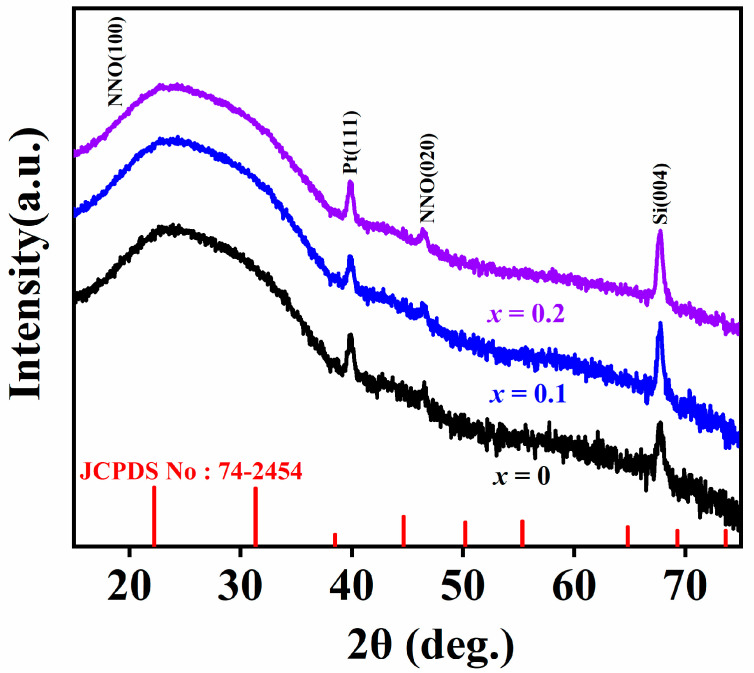
XRD patterns for (1−*x*)NN-*x*CT thin films on (111) Pt/Ti/SiO_2_/Si substrates.

Figure 2 displays the XRD pattern of the (1−*x*)NN-*x*CT film deposited on the (111)Pt/Ti/SiO_2_/Si substrate. The positions of the film characteristic peaks are consistent with the standard PDF card (JCPDS No: 74-2454) [22], demonstrating a polycrystalline perovskite structure. At the same time, the incorporation of smaller A-site (Ca^2+^)- and larger B-site (Ti^4+^)-doped ions almost counteracts the effects of lattice expansion and contraction, resulting in negligible changes in lattice parameters in the NN films.

**Figure 3:**
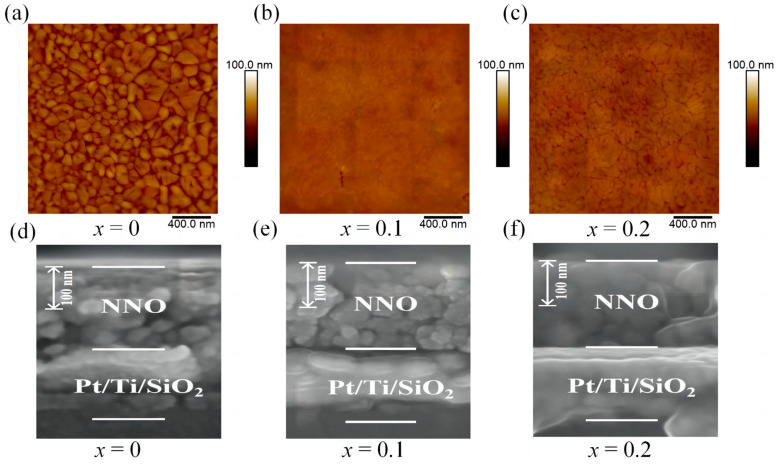
AFM and cross-SEM images of (1−*x*)NN-*x*CT thin films. (**a**,**d**) *x* = 0, (**b**,**e**) *x* = 0.1, (**c**,**f**) *x* = 0.2.

Figure 3a–c presents the AFM images of (1−*x*)NN-*x*CT thin films with a scan size of 2 μm × 2 μm. The CT content highly affected the surface morphologies. Figure 3a shows un-doped NN thin film with a rough surface, while CT-doped NN thin films in Figure 3b,c exhibit the properties of denser surface and smaller grains. As well known in chemical-solution-derived niobate-based thin films [13,23,24], the suppression of grain growth leads to a decreasing grain size. This suppression is likely due to the decreased volatilization of Na^+^ and a reduction in oxygen vacancies within the crystal, which affects the mass transfer between grain boundaries and inhibits the growth of grains.

Moreover, the root mean square (RMS) of surface roughness, determined through Igor Pro software (Igor Pro 6.37), is approximately 4.7 nm, 1.1 nm and 1.9 nm for *x* = 0, 0.1, 0.2, respectively. RMS values decrease and then increase with the increase of *x*, reaching a minimum at *x* = 0.1. Z. Song et al. [25] reported that grain size is inversely proportional to the breakdown strength of dielectric material, and smaller grains are more favorable for energy storage. Figure 3d–f show the cross-sectional images of thin films with different CT contents, all of which maintain a consistent thickness of approximately 200 nm.

**Figure 4:**
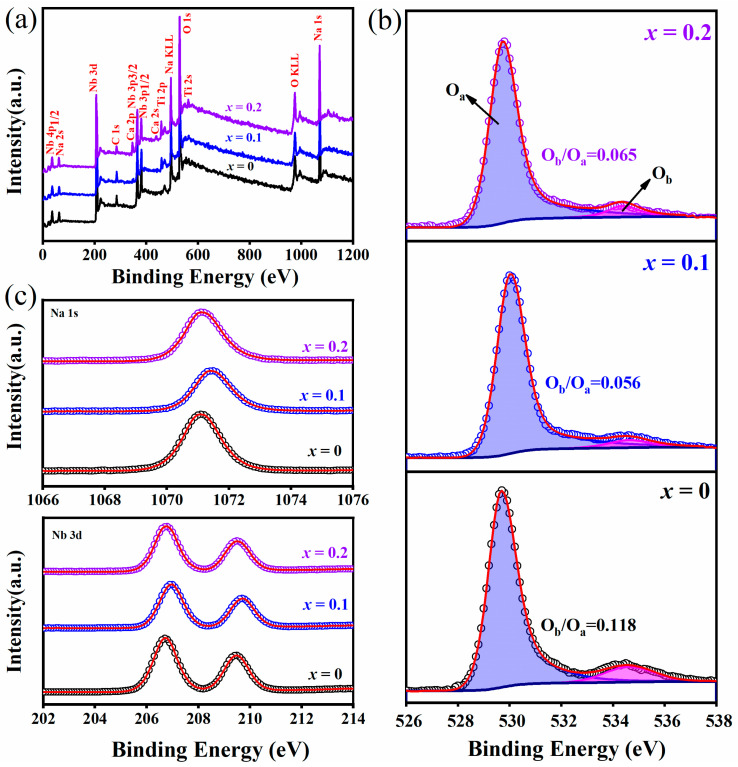
XPS spectra of (1−*x*)NN-*x*CTthin films: (**a**) XPS survey spectra, (**b**) relative area ratios of O_b_/O_a_ as a function of *x*-level, (**c**) narrow scan Na 1s and Nb 3d XPS spectra.

The XPS measurement was employed to identify the constituent elements in (1−*x*)NN-*x*CT thin films. The main characteristic peaks in XPS full spectra, as shown in Figure 4a, support the existence of the expected elements, i.e., Na, Nb, O, Ca and Ti. Figure 4b shows the high-resolution XPS spectra of the O1s region for (1−*x*)NN-*x*CT thin films. The three sub-figures display two distinct peaks due to their asymmetry, corresponding to the lattice oxygen (O_a_) and oxygen vacancies (O_b_), respectively [26]. The ratio of peak area O_b_/O_a_, i.e., indicating the relative quantity of oxygen vacancies, were 0.118, 0.056 and 0.065 for *x* = 0, 0.1 and 0.2, respectively. This means that the relative density of oxygen vacancies initially increases and then decreases with *x*. The possible explanation is that the volatilization of Na^+^ decreases with increasing CT content, resulting in a reduction in oxygen vacancies in the thin films. When *x* = 0.2, the increase in oxygen vacancies could be attributed to the decrease in the internal density of the sample and the increase in pores in the thin film [27]. Additionally, the elements of Na and Nb present fixed valence, i.e., Na^+^ and Nb^5+^, as presented in Figure 4c.

**Figure 5:**
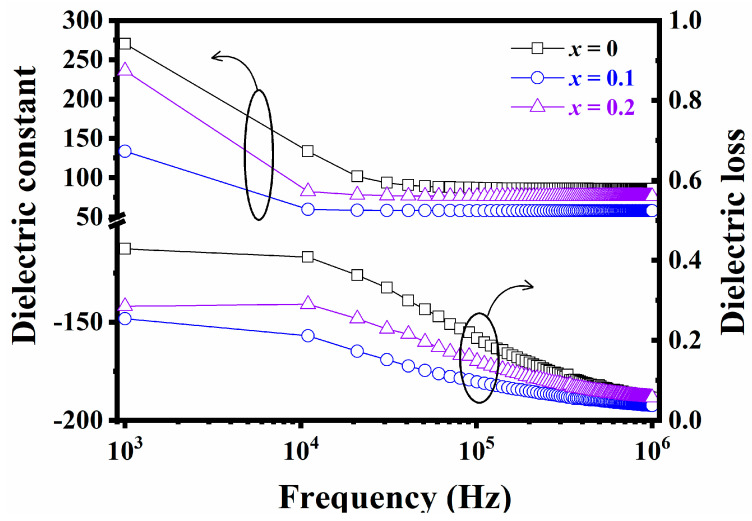
Frequency—dependent dielectric constant (three curves surrounded the black circles with left arrows) and loss (three curves surrounded the black circles with right arrows) of (1−*x*)NN-*x*CT thin films.

The frequency-dependent dielectric constant and loss of (1−*x*)NN-*x*CT thin films are illustrated in Figure 5. It is evident that the dielectric constant decreases with increasing frequency, resulting from the dipoles unable to follow the conversion of the external electric field within the frequency increasing and reflecting the relaxation behavior. In addition, the decrease initially and then increases in dielectric constant at the same frequency with increasing x-level. The variation of the dielectric constant is strongly connected with grain size of the thin films, as the phenomenon frequently observed in dielectric thin films [28,29]. With the increase of *x*, the dielectric loss has the same trend as the dielectric constant, reaching a minimum at *x* = 0.1, which is mainly due to the decrease in the number of oxygen vacancies in the thin films.

**Figure 6:**
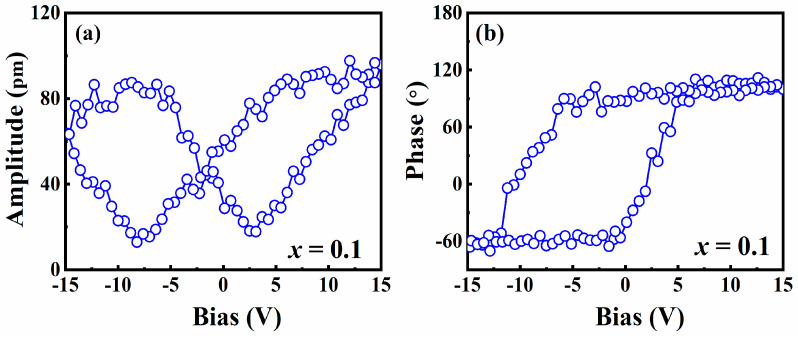
Amplitude hysteresis loops (**a**) and local phase (**b**) acquired in the 0.9NN-0.1CT thin film.

The thin film with *x* = 0.1 was chosen to measure the piezoelectric hysteresis loops to characterize the local electric properties of the (1−*x*)NN-*x*CT thin films. Figure 6a,b illustrate the piezoresponse phase—voltage hysteresis and the butterfly-like amplitude-voltage loops, respectively. The rather stable amplitude and phase exhibit a low bias, indicating no reversal process. Upon increasing the bias voltage to 15 V, the thin film exhibits a well-butterfly amplitude loop and a square phase hysteresis loop, indicating its ferroelectric characteristic. Furthermore, the hysteresis and butterfly loops depicted in Figure 6 exhibit asymmetrical distributions. The asymmetrical phenomenon in the PFM hysteresis loop may be attributed to the built-in fields resulting from the work—function difference between the top/bottom electrodes and the NN [30].

## 4. Conclusions

In summary, CT-doped NN thin films were grown on (111) Pt/Ti/SiO_2_/Si substrates via the sol-gel method, and the effects of different CT doping concentrations ((1−*x*)NN-*x*CT, *x* = 0, 0.1, 0.2) on the microstructure and electrical properties were investigated. The XRD spectra confirm that all thin films exhibit perovskite structure, without any detectable impurity phases. Notably, as *x* increases, a reduction was observed in both grain size and surface roughness, reaching a minimum at *x* = 0.1. That is, the surface is denser and more uniform at *x* = 0.1. The results of XPS and dielectric properties show that the number of oxygen vacancies and dielectric loss decrease and then increase with the increase of *x*, with a minimum observed at *x* = 0.1. The above study indicates that the thin film has the optimal microstructure, oxygen vacancy content and dielectric properties when the CT doping content is 0.1. Afterwards, the PFM properties of the 0.9NN-0.1CT thin film were characterized, revealing well-defined phase hysteresis and amplitude butterfly loops with obviously ferroelectric properties. The obtained results lay a solid groundwork for the subsequent implementation of NaNbO_3_ in energy-related applications.

## Data Availability

The data presented in this study are available on request from the corresponding author.
